# New Statistical Approach to Apportion Dietary Sources of Iodine Intake: Findings from Kenya, Senegal and India

**DOI:** 10.3390/nu10040430

**Published:** 2018-03-29

**Authors:** Frits van der Haar, Jacky Knowles, Zipporah Bukania, Boubacar Camara, Chandrakant S. Pandav, John Maina Mwai, Ndeye Khady Toure, Kapil Yadav

**Affiliations:** 1 Rollins School of Public Health, Emory University, Atlanta, GA 30322, USA; 2 Large Scale Food Fortification Initiative, Global Alliance for Improved Nutrition, 1211 Geneva, Switzerland; jacky@jackyknowlesconsultancy.com; 3 Center for Public Health Research, Kenya Medical Research Institute, 00202 Nairobi, Kenya; zbukania@gmail.com; 4 Comité Scientifique de l’Ecole Doctorale, Université Cheikh Anta Diop de Dakar, B.P. 5005 Dakar-Fann, Sénégal; bccamara@yahoo.com; 5 All India Institute of Medical Sciences, Iodine Global Network, New Delhi 110029, India; cpandav@iqplusin.org; 6 Ministry of Health, Nutrition and Dietetics Unit, P.O. Box 43319-00100, Nairobi, Kenya; kiriromwai@gmail.com; 7 Cellule de Lutte contre la Malnutrition, B.P. 45001 Dakar-Fann, Sénégal; ndeyekhadytoure@yahoo.fr; 8 Centre for Community Medicine, All India Institute of Medical Sciences, New Delhi 110029, India; dr.kapilyadav@gmail.com

**Keywords:** universal salt iodization, native dietary iodine, processed foods, household salt, population iodine intake apportionment

## Abstract

Progress of national Universal Salt Iodization (USI) strategies is typically assessed by household coverage of adequately iodized salt and median urinary iodine concentration (UIC) in spot urine collections. However, household coverage does not inform on the iodized salt used in preparation of processed foods outside homes, nor does the total UIC reflect the portion of population iodine intake attributable to the USI strategy. This study used data from three population-representative surveys of women of reproductive age (WRA) in Kenya, Senegal and India to develop and illustrate a new approach to apportion the population UIC levels by the principal dietary sources of iodine intake, namely native iodine, iodine in processed food salt and iodine in household salt. The technique requires measurement of urinary sodium concentrations (UNaC) in the same spot urine samples collected for iodine status assessment. Taking into account the different complex survey designs of each survey, generalized linear regression (GLR) analyses were performed in which the UIC data of WRA was set as the outcome variable that depends on their UNaC and household salt iodine (SI) data as explanatory variables. Estimates of the UIC portions that correspond to iodine intake sources were calculated with use of the intercept and regression coefficients for the UNaC and SI variables in each country’s regression equation. GLR coefficients for UNaC and SI were significant in all country-specific models. Rural location did not show a significant association in any country when controlled for other explanatory variables. The estimated UIC portion from native dietary iodine intake in each country fell below the minimum threshold for iodine sufficiency. The UIC portion arising from processed food salt in Kenya was substantially higher than in Senegal and India, while the UIC portions from household salt use varied in accordance with the mean level of household SI content in the country surveys. The UIC portions and all-salt-derived iodine intakes found in this study were illustrative of existing differences in national USI legislative frameworks and national salt supply situations between countries. The approach of apportioning the population UIC from spot urine collections may be useful for future monitoring of change in iodine nutrition from reduced salt use in processed foods and in households.

## 1. Introduction

Universal Salt Iodization (USI) is the preferred strategy for preventing iodine deficiency disorders (IDD) [1,2]. After iodizing the entire production of food-grade salt [3], iodized salt becomes an intrinsic part of the common diet [4] and provides iodine intake by way of the salt used during cooking and seasoning of meals in the households (referred to in this paper as household salt) and the salt used in processed foods obtained from outside the homes (referred to as processed foods). The intakes of iodine from these distinct types of salt add to the iodine intake from naturally present iodine in foods and liquids (referred to as native iodine) that make up the common diet. Together with native iodine, the two iodine sources from household salt and processed foods aim at reaching a combined iodine intake that meets biological iodine requirements [5]. For assessing the outcome of a particular USI strategy and considering whether adjustment of an iodized salt standard may be needed, it is important to have information about the iodine intakes attributable to native iodine and the two salt-derived dietary iodine sources.

The percent of households using iodized salt is a convenient benchmark of USI progress [6] and there has been a quantum leap in global household coverage of adequately iodized salt [7] since the IDD elimination goal was adopted in 1990. Nevertheless, assessment of USI achievement from the household coverage alone does not capture the full reach of a true USI strategy because it does not inform on the iodized salt that was used in consumed processed foods and in the preparation of foods obtained outside the home.

Urinary iodine concentration (UIC) is a reliable biomarker of recent iodine intake because more than 90% of ingested iodine is excreted in urine within 24–48 h [8]. WHO recommends assessment of population iodine status from spot urine UIC measurements [6] and by consensus, a median UIC of 100–299 µg/L in school-age children (SAC) or 100–199 µg/L in non-pregnant adults indicates adequate iodine nutrition among that population [2]. However, because UIC captures the total iodine intake, population iodine nutrition assessments based on the median UIC do not reveal information about the iodine intake that can be attributed to the USI strategy.

Commonly available “on-the-spot” rapid salt test kits do not provide reliable data of the salt iodine content in field settings [9]. Therefore, population surveys have gradually started collecting salt samples from the households to measure the iodine content for assessment of USI progress and analysis of the contribution by iodized household salt to population iodine nutrition. However, comparisons across populations between findings of median UIC and household salt iodine content or coverage are typically difficult to interpret when information on the native-source dietary iodine intake and the contribution of iodized salt from processed foods is lacking.

In countries where salt iodization is well-established, the excretion of iodine in urine closely reflects the consumption of iodized salt [2,8,10]. Indeed, in populations with limited access to processed foods, the iodine content of household salt is closely associated with UIC levels [11,12,13]. Because the volume of urine depends on water intake [14,15], concentrations of iodine as well as sodium in urine are co-determined by the intake of fluids, and as the urinary iodine and sodium concentrations respond in the same way to variations in urine volume, concurrent urinary iodine and urinary sodium concentrations can be expected to fluctuate in tandem [16]. Concentrations of iodine and sodium measured in spot urine samples of healthy individuals are therefore likely to show an association.

In view of the dependency of the UIC on recent iodine intake, we conjectured that the urinary sodium concentration (UNaC) could provide useful information for the interpretation of UIC variations related to the amount of iodized salt in the diet. Measuring the concentration of sodium in urine samples already collected for iodine nutrition assessment puts little additional burden on the field workers and participants. However, the sodium assay adds to the laboratory costs, which may turn out to be less affordable in low-resource settings.

The present study set out to develop and illustrate a statistical technique to apportion the key sources of iodine intake, namely native iodine, processed foods and household salt, from UIC and UNaC measurements in spot urine samples, combined with the iodine content (SI) in salt samples from the households of the survey target group.

## 2. Materials and Methods 

Data for the present study were from three population-representative national iodine nutrition surveys of non-pregnant women of reproductive age (WRA) in Kenya (2011), Senegal (2015) and India (2014–2015). SI and UIC findings from these surveys have been reported [17,18,19]. For each survey, ethical approvals were granted by the respective national authorities. In Kenya, the data collection formed part of a multi-purpose micronutrient survey while the data in Senegal and India were obtained during a dedicated iodine nutrition survey. In each country, a single spot urine sample from WRA and a salt sample from the household of WRA were collected during daylight hours. Demographic and food use information of WRA and her household were recorded concurrently. 

Survey tools in Senegal and India included a questionnaire module to assess household vulnerability to poverty with a validated Multi-Dimensional Poverty Index (MPI) score [20], which consisted of three dimensions: Educational attainment and school attendance (MPI Education); Food security, hunger and child mortality (MPI Health); and Sanitation, water, fuel, electricity, floor and household assets (MPI Living Standards). The MPI score estimates the extent of household vulnerability to poverty, or deprivation; a higher overall MPI score corresponds to higher vulnerability and a score of >0.3 within each dimension indicates deprivation in that dimension. Because bouillon seasoning is common in Senegal [21], the survey questionnaire in Senegal included questions on bouillon consumption during the week prior to urine collection.

### 2.1. Laboratory Methods

Salt and urine samples after collection were kept cool and dark until delivery to a central laboratory where the urine samples were stored frozen at −25 °C until analysis. In each country, salt iodine (SI) content in mg/kg was obtained by iodometric titration [22] and UIC (in µg/L) was analyzed by the Sandell-Kolthoff reaction after digestion with ammonium persulphate [23,24]. In Kenya, UNaC (in mmol/L) was assayed by atomic absorption spectrophotometry and in Senegal and India, UNaC was obtained with an ion-sensitive electrode. Each laboratory applied strict internal quality control procedures. During the period of UIC analysis, the laboratories in Kenya and India obtained a certificate of successful participation from the Program to Ensure the Quality of Urinary Iodine Procedures (EQUIP) of the U.S. CDC in Atlanta, U.S.A. To assess the accuracy of UIC and SI results from the Kenya laboratory, a series of blind duplicate urine and salt samples were analyzed in two reputed laboratories in Tanzania and Kazakhstan. Comparisons of UIC results indicated identical measurement performance in each laboratory. The SI results of the Kenya laboratory were systematically elevated by 6.9 mg/kg from findings in the same salt samples by the two external laboratories however. For comparability, the SI data in this report were corrected for this difference prior to statistical analyses. To ensure the quality of SI and UIC analyses results within acceptable limits, the laboratories in India and Senegal collaborated with an external service laboratory that provided internal salt and urine control samples with known iodine content prior to the surveys, and several rounds of salt and urine samples with unknown iodine content before and during the period of urine and salt analyses. Once the reported values of unknown external salt and urine samples in a given laboratory were within limits for acceptance, i.e., below 10% variation from values predetermined in the service laboratory, the data of survey sample analyses were released.

### 2.2. Kenya

The iodine component of the Kenya National Micronutrient Survey (KNMS) aimed to obtain representative estimates of the iodine status of the population nation-wide and by urban and rural area. The design consisted of two-stage stratified cluster sampling with use of the National Sample Survey and Evaluation Program IV master frame, maintained by the Kenya National Bureau of Statistics. A total of 296 clusters were drawn proportionate-to-population size from the master frame, resulting in 123 urban and 173 rural primary sampling units (PSU’s). In each PSU, 4 households were drawn at random from especially prepared exhaustive household lists for enrollment of all residing WRA. Urban areas were defined to encompass cities, municipalities, town councils, urban councils and all district headquarters, while rural areas were defined as all isolated large areas of open country with people whose main economic activity was farming. The survey protocol was reviewed and approved by the Ethical Review Committee of the Kenya Medical Research Institute and all WRA who agreed to participate provided written informed consent. The participants did not receive a monetary incentive.

### 2.3. Senegal

The Senegal National Iodine Survey 2015 aimed to provide estimates of the household iodized salt coverage at national level and disaggregated by three strata, defined as urban, rural small-scale salt-producing, and rural-non-salt-producing. The choice of stratification was agreed on in order to obtain representative data on which to base adjustment and prioritization of the national USI strategy. A listing of salt-producing and non-salt-producing districts was provided by Cellule de Lutte contre la Malnutrition (CLM) to the Agence Nationale de Statistiques et Démographie who applied population-proportionate sampling within each stratum using the 2013 population census listing of enumeration areas. The optimal sampling structure was determined as 41 PSU’s in each of the three strata. Segmentation of approximately equal segments was conducted where the enumeration area was particularly large, followed by random selection of one segment for inclusion in the survey sample. Exhaustive lists of households were prepared for all the 123 PSU’s, followed by systematic selection of 16 households within each PSU. Samples of household salt of approximately 50 g were collected from all the households within each PSU. The intent was to enroll one woman of reproductive age wherever they were present in a selected household. From demographic and likely response rate information, the number of non-pregnant women expected in the final survey sample was 1350. One WRA from the household was asked about her frequency of consumption of foods containing bouillon over the previous week (number of days when consumed and average times per day) and this was used to represent typical bouillon consumption patterns for all WRA in the household. The survey protocol was reviewed and approved by the Comité Nationale d’Etique du Sénégal. The survey participants did not receive a monetary incentive.

### 2.4. India

The India National Iodine Survey 2014–2015 aimed to provide estimates of the household iodized salt coverage at national level and disaggregated by urban and rural residence in six geographical zones. Zones were defined according to the State Reorganization Act 1956 and covered all the present States and Union Territories of India. The optimal sampling structure was determined as 42 PSU’s each in the urban and rural strata of each zone. Population-proportionate sampling with the use of the 2011 population census was applied to for the selection of 42 wards (urban PSU’s) and 42 villages (rural PSU’s) in each of the 6 zones. Exhaustive lists of households were prepared for all the 504 PSU’s, followed by systematic selection of 12 households within each PSU. Household salt samples of approximately 50 g were collected from all the households within each PSU but spot urine samples were obtained from all consenting WRA present in alternate households only. The Ethics Committee of the All India Institute of Medical Sciences, New Delhi, India, provided ethical approval and informed written consent was obtained from each survey respondent and WRA prior to the salt and urine sample collection respectively. The households received a small monetary compensation for providing the salt sample.

### 2.5. Data Analyses

Descriptive findings for SI, UIC and UNaC in each survey were obtained with SPSS version 22. Combined analyses of the UIC, UNaC and SI data with Stata 14 (StataCorp LP, College Station, TX, USA) used an extension of simple linear regression between urinary iodine (dependent variable) and urinary sodium findings (explanatory variable) in Swiss adults [25]. To take into account the complex survey design, which differed in each survey, generalized linear regression (GLR) analysis [26] treated the UIC data of WRA as an outcome variable that is dependent on their UNaC and SI data as explanatory variables. Other explanatory variables, i.e., rural location, bouillon consumption and MPI scores, as well as interactions were added as and when appropriate. All regression analyses were performed with ^e^log-transformed UIC data to reduce skewness of the raw data distributions, and survey weights were used to account for non-response and missing data. Aptness of the GLR models was assessed graphically, homoscedasticity was examined by plots of the standardized residuals against predicted ^e^logUIC values and the normality of residuals was checked with probability plots. For the datasets from Kenya and India, 95% confidence intervals were calculated without finite population correction as the population size of each country is very large compared with the sample sizes of WRA. For the processing of data from Senegal, the number of PSUs in each stratum and the total number of households in each selected PSU were used as finite population corrections. ^e^LogUIC findings from GLR analyses were back-transformed to the original µg/L scale and, therefore, the reported UIC portion estimates are geometric UIC mean values.

UIC portion estimates that correspond with the dietary sources of iodine intake were calculated with use of the intercept and regression coefficients of UNaC and SI variables from each country’s regression equation, while entering the mean findings for any other explanatory variables at each appropriate step. First, the intercept estimate was used to calculate its back-transformed UIC value, which is the geometric mean UIC level that does not depend on either the UNaC or the SI value as both are zero. The resulting finding is interpreted as the UIC portion attributable to native iodine intake. Second, the back-transformed UIC value was calculated with the regression equation while inputting the average UNaC finding of WRA and holding the SI content at zero. The resulting UIC estimate therefore corresponds with the mean UNaC finding that originated from all dietary salt with zero iodine content in the household salt fraction. The difference between this UIC estimate and the former UIC value (calculated from the intercept alone) is interpreted as the UIC portion due to iodine intake from processed foods. Finally, the difference between the total UIC and the UIC estimate arising from dietary salt (the 2nd step UIC calculation) is interpreted as the UIC portion attributable to iodine intake from household salt.

## 3. Results

For the present report, the number of WRA in each country was restricted to those with a complete set of data for analysis.

### 3.1. Descriptive Findings

Table 1 shows descriptive findings of the WRA in Kenya, Senegal and India. SI contents (mean, SD) in Kenyan households were 34.4 ± 18.3 mg/kg, in Senegalese households 14.9 ± 13.0 mg/kg, and SI levels in Indian households were 25.7 ± 16.9 mg/kg. Not surprisingly, findings for household coverage with adequately iodized salt (≥15 mg/kg) varied in accordance with the SI findings: 94.9% in Kenya, 37.1% in Senegal and 78.1% in India. Using recommended guidelines [6], median UIC findings in WRA of 172 µg/L in Kenya, 159 µg/L in India and 101 µg/L in Senegal were indicative of adequate population iodine nutrition, though the median UIC of WRA in Senegal barely reached above the 100 µg/L minimum threshold for iodine sufficiency. The geometric mean UIC findings of 179 µg/L in Kenya, 138 µg/L in India and 92 µg/L in Senegal reflected significant positive skewness of the UIC data distributions in Senegal and India. Across countries, UNaC findings of WRA (mean, SD) were highest in Kenya (185 ± 97 mmol/L), followed by Senegal (167 ± 80 mmol/L) and lowest UNaC values (140 ± 69 mmol/L) were found in India. SI and UIC findings in each country were higher in urban than rural areas, but differences across countries in UNaC findings between the urban and rural WRA were variable. The WRA in urban Kenya had higher UNaC than their rural counterparts; UNaC findings differed little between the urban and rural areas in Senegal, while WRA in rural India had slightly higher mean UNaC levels than their urban peers.

### 3.2. Analytical Findings

Results from the multiple GLR analyses by country are reported in Table 2. Intercept and slope estimates for the UNaC and the SI variables were statistically significant for all country-specific models and the associations between the UIC and UNaC values of WRA were particularly strong. Rural household type did not show a significant association with the UIC in any country when controlled for the other explanatory variables. In India, MPI score for Living Standards showed a significant association with the UIC, indicating that WRA from deprived households, controlled for all other explanatory variables, had lower UIC than their counterparts. Inputting mean values for the other variables, UIC levels of WRA from deprived households were ±10 µg/L lower than of WRA from non-deprived households. For Senegal, neither the MPI scores nor the self-reported bouillon consumption of WRA showed a significant independent association with UIC levels.

### 3.3. Estimation of UIC Portions That Correspond to Dietary Sources of Iodine Intake

**Figure 1:**
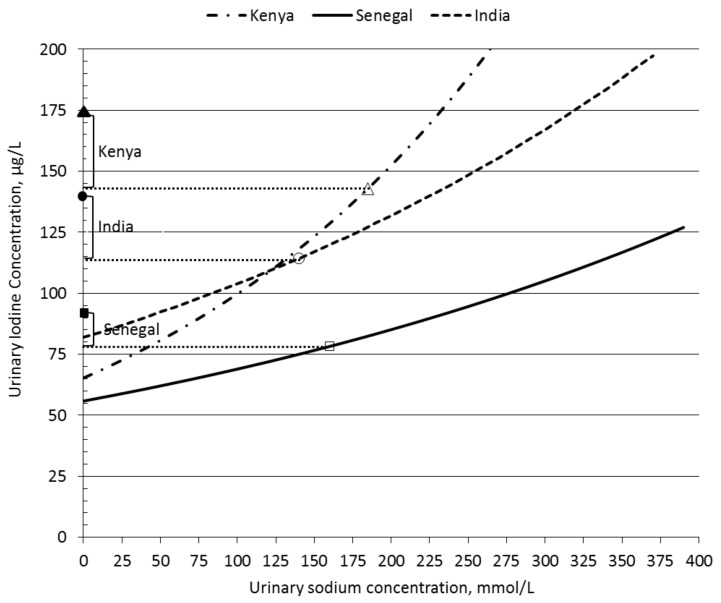
Estimation of UIC portions corresponding with dietary sources of iodine intake of WRA. Marker symbols: Kenya triangles, Senegal squares, India circles. Open symbols indicate the location of average UNaC findings in each country. Their corresponding UIC levels are shown by the horizontal dotted lines. Closed symbols show the total UIC findings and UIC portion estimates attributable to iodine intakes from household salt are illustrated by square brackets.

Figure 1 illustrates the step-wise approach of estimating the dietary iodine source-related UIC portions with the GLR coefficients reported in Table 2. The abscissa (Y axis) of UIC values in Figure 1 is shown in back-transformed µg/L units. The Figure depicts the side face of UIC and UNaC relationships from the three-dimensional space of regression planes, defined by the country-specific associations between the UIC values of WRA and their UNaC and SI values. The rising lines in Figure 1 display the country-specific regression equations for the association between UIC and UNaC values, controlled for all other explanatory variables. As initially conjectured, the UIC values of WRA show increases with the rise of UNaC values in each country. UIC portions due to native dietary iodine intake are found at the point where the regression lines for each country cross the abscissa. Open marker symbols plotted on the regression lines show the expected UIC levels at the average UNaC findings of WRA in each country, and their corresponding UIC values are indicated by the horizontal dashed lines. Estimates for the food salt-source UIC portions in each country are derived as the difference with the UIC portion calculated from the intercept alone. Finally, the (geometric) mean total UIC findings of WRA in each country are plotted on the abscissa by their respective closed marker symbols and estimates for the household salt-sourced UIC portions (shown by the square brackets in Figure 1) are calculated as the difference with the UIC finding that corresponded with the average UNaC finding, i.e., the result of the previous step).

Associations between the UIC and UNaC values of WRA (Figure 1) show a clearly steeper rise of UIC in the Kenyan situation than in Senegal and India at the comparable UNaC range. Further analysis of the differences in slope (details not shown) found a significantly greater partial regression estimate for the relationship between the UIC and UNaC values of Kenyan WRA than in Senegal and India (*p =* 0.002 and *p* = 0.001 for the comparison with Senegal and India, respectively). In contrast, the country-specific slope estimates for the associations between the UIC and SI values, controlled for other variables, did not differ statistically.

### 3.4. Comparison between Countries

**Figure 2:**
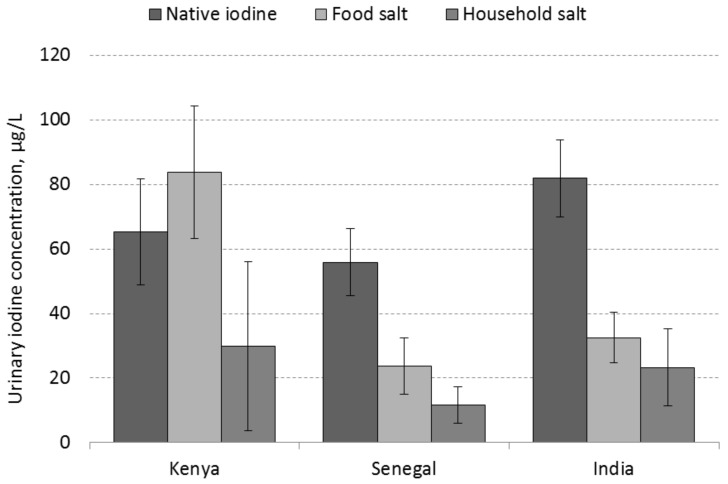
UIC ^1^ portions (geometric mean, 95% CI) by dietary source of iodine intake of WRA in Kenya, Senegal and India. **^1^** UIC = Urinary iodine concentration, WRA = Women of reproductive age.

Country-specific UIC portions that correspond with the different sources of iodine in the diet of WRA are reported in Table 3. Figure 2 shows the mean UIC portions by country and their 95% CI estimates. Notably in each country, the UIC portion estimates that reflect the native dietary iodine intake were well below the 100 µg/L minimum threshold for iodine sufficiency. The UIC portion arising from processed food salt in Kenya was starkly higher than in Senegal and India, while the UIC portions from use of iodized household salt were consistent with the differences in mean SI content of household salt across countries. UIC portion estimates that correspond with native iodine, iodized food salt and iodized household salt in Kenya were 36%, 47% and 17%, respectively, of the total UIC of WRA. These proportions in Senegal were 61%, 26% and 13%, and in India 59%, 24% and 17%, respectively. 

## 4. Discussion

The method of estimating UIC portions that correspond with the sources of iodine intake may be particularly valuable in view of the difficulties in measuring the consumption of iodine and salt with dietary methods. Due to a dependence on iodine content in soil and ground water [27,28], the iodine content in common foods can vary considerably by geography [29], which makes an accurate estimate for native dietary iodine intake, i.e., without iodized salt, problematic. Seasonal variations in iodine content of some food items, such as dairy products [30], and the few reliable iodine data in food composition tables [29,30,31] are additional difficulties in studies of iodine consumption with dietary methods. A review of this topic has been published very recently [32]. Quantifying the discretionary intake of salt, whether iodized or not, is also challenging [33]. With increasing industrialization, an ever greater share of a population’s salt intake comes from processed foods [34] and even while the sodium content in processed food may be cited in food tables, they seldom specify whether the salt used in the recipes of processed foods is iodized or not, and if so, at what level. Measuring the consumption of iodine or salt with dietary methods is, therefore, not easy.

Use of the median UIC from a large number of spot urine samples is a time-tested practice for assessing and classifying population iodine intake [31]. Recently, a promising approach has emerged to use the UIC data distribution after adjustment for individual variation to estimate the prevalence of deficient and excess iodine intakes [31,35]. Use of UNaC measurements in casual urine samples for estimating population salt intake is considered less reliable, however [36,37], and a recent nomenclature for classifying population salt intakes does not include the UNaC [38]. Accordingly, the UNaC data in this report were not directed to estimate the salt intake but to serve as an iodine intake-associated data source for separation of the UIC levels by the salt and non-salt origins of iodine intake.

The percentage of households using adequately iodized salt is an accepted measure for the reach of USI strategies [6,8,39]. In areas with limited or no access to commercially processed foods, the response in UIC level to an increase in household coverage is consistent over time and mirrors the iodine content [8,40]. This dose-response relationship suggests that the SI content in such conditions closely reflects the iodine intake from iodized salt use in the household. It is important, however, to realize that SI measurements do not reveal the amount of iodized household salt actually consumed, nor does the SI variable reflect the amount or quality of iodized salt consumed in snacks, street foods, meals in restaurants and worker canteens, or other types of foods eaten outside the home.

Multiple regression analysis is a common technique to estimate the relationship of an outcome variable with a set of predictor variables [41]. Epidemiologic studies often use regression to estimate risk based on information from risk predictors, but contrary to the goal of predicting risk for specific people, much of the epidemiologic regression research is also aimed at learning the explanatory role of specific factors in health or disease [42]. The latter was the purpose of this study of relationships between the iodine status and the household IS content and urinary sodium data of the WRA cohorts. Because linear regression was the prominent analysis tool in this study, it is important to review some details of this technique. A regression coefficient, i.e., slope, can be biased when the constituent variable differs from sample to sample [43,44]. Individual variability in the explanatory variable, particularly the UNaC, diminishes the magnitude of the slope [45] and increases the intercept, derived from the slope. The extent to which in this study a reduction of slope from existing variability of the spot UNaC data [36] did result in a higher UIC intercept estimate cannot be known, but it is acknowledged that the reported findings for native dietary iodine intake are likely higher than their true values. Individual variability of the outcome variable, i.e., the UIC in this study, also affects the slope [45] but it does so by increasing the 95% CI, which means higher uncertainty of all the UIC portion point estimates. Two major underlying causes for the inherent variability of UIC and UNaC are variations in food consumption patterns and body hydration. Nevertheless, because the regression technique uses least-squares minimization [41,43], the estimates for UIC portions that correspond with the mean iodine intake from the two types of dietary salt are likely not affected. Taken together, these statistical considerations would suggest that the use of spot UNaC data has likely inflated the native iodine intake estimates and, thereby, decreased those for iodine intake with processed food salt, while the use of spot UIC data in regression analysis is a likely cause for higher uncertainty of all the UIC portion point estimates.

Especially in rural areas of less-industrialized countries, consumers do not often purchase what are considered processed foods in Western societies but only minimally processed foods, such as bread and biscuits, which are mostly sold without packaging or branding. An exception in this study was bouillon, a widely marketed and packaged seasoning product used in preparing meals in the households and a significant source of salt intake in Senegal [46]. The multiple regression findings for Senegal, however, did not yield a significant independent association for self-reported bouillon consumption with UIC. Lack of a significant association in this case may be explainable from differences in iodine content between various brands of bouillon cubes and sachets, or by limited emphasis in the questionnaire on assessing the possible short-term effect, i.e., within the past 24–48 h, from bouillon consumption on the UIC.

From an expectation that household access to adequately iodized salt and, therefore, the iodine status of WRA would differ, each survey adopted the common practice of distinct sampling in urban and rural areas. Indeed, as Table 1 shows, the UIC findings of urban WRA exceeded those of their rural counterparts in each country. The findings of dependency of the UIC levels on the UNaC, SI and residence variables (Table 2), however, showed that rural household location, controlled for all other variables, was not a significant explanatory factor in any country. In Kenya, the higher UNaC and SI findings of urban WRA and their households would account for the observed urban-rural divide in iodine status. In Senegal, the regression coefficient for the SI variable indicated a much stronger dependency of the UIC on SI content than in the two other countries. The SI finding in urban areas was twice as high as in the rural cohorts combined, which suggests that the SI level of household salt was the major defining factor for the observed urban-rural difference in iodine status of the Senegalese WRA. In India, where the UNaC findings of urban and rural WRA were comparable, the different SI levels between urban and rural households alone would not suffice to explain the existent urban-rural divide in iodine status of WRA. We speculate that the WRA in rural India may have consumed a higher amount of household salt and less processed foods than the WRA in urban centers. This issue deserves further study in India.

Findings for the salt-source UIC portions illustrate the differences that exist in legislative frameworks and salt production and supply situations between the countries in this study. Legislation in Kenya and Senegal compel the salt industry to supply only iodized salt for all human consumption purposes, while the USI strategy in India rests on a nationwide ban on the sale of non-iodized salt in consumer markets. Industry standards for iodized salt in Kenya and in Senegal are set at 30–50 mg/kg, while in India, a minimum of 30 mg/kg is required for the supply of iodized salt to consumers, but salt producers are not obligated to sell iodized salt to their customers in food industry. The national salt supply in Kenya is consolidated between three large-scale salt producers along the Indian Ocean coast [47], each of which have achieved a premium quality mark for their salt supplies from the close collaboration in supportive oversight with the Kenya Bureau of Standards. Findings of SI content and household coverage in Kenya demonstrated evidence of true USI, which was manifested in a combined salt-source UIC portion estimate of 114 µg/L, or 64% of the total iodine intake of WRA. To compare, the salt supply situation in Senegal is highly dispersed over several clusters of numerous small producers who typically are facing serious quality assurance and control challenges [48]. Findings of SI content and household coverage in Senegal demonstrated a considerable shortfall of USI progress, which was reflected in a combined salt-source UIC portion estimate of only 36 µg/L, or 39% of the total iodine intake. As the USI strategy in India is only binding on consumer markets, the responsibility for enforcement falls under the regular local food control authorities. About 80% of all food-grade salt in India is produced in large- and medium-size companies, but this leaves more than 1 million metric ton of salt per year to numerous small-scale producers who are struggling with identified quality assurance and control issues [48]. Findings of SI content and household coverage in India, though falling short of USI targets, exceed those in Senegal and are reflected in a combined salt-source UIC portion estimate of 56 µg/L, or 41% of the total iodine intake. Compared with Kenya, the relatively small UIC portion from processed foods in India is more likely due to a default use of iodized salt intended for household use by local, small-scale processed food preparers, rather than deliberate purchases by large-scale food processing industries.

A limitation of approach in this study is that the native-source sodium was not taken into account in estimating the salt source-related UIC portions. Studies of common diets of the UK [49] and the US [50], and a recent analysis of salt and iodine intake in Swiss adults [25] have reported that 12–18% of the sodium consumption in these Western countries originates from native sodium content in foods. We could not identify a similar estimate for less industrialized countries. Native dietary sodium may contribute somewhat less to the total sodium intake in the countries of this study where cereals (maize, rice), vegetables and pulses are dominant common foods. Nevertheless, a procedure that estimates the UIC portions with use of the mean UNaC after correction for native-source dietary sodium would have allowed more accurate estimates for the native and processed food salt UIC portions in each country.

The strength of this study would be the illustration of an eminently operable method to approximate the sources of iodine intake with data from large house-to-house surveys with no additional burden on the respondents and only a small additional expenditure for sodium analysis in the urine samples already collected for iodine assessment. Practical advantages of spot urine sampling over 24 h collections have been well described [51] and recognition has grown that the average UNaC from a large number of spot urine collections can approximate the mean daily sodium excretion well enough for the purpose of monitoring changes in the sodium intake of populations over time [52,53]. The current study estimated the salt-source UIC portions by way of the average UNaC value in spot urine samples—a procedure that may also be useful for future monitoring of change in population iodine intakes from changes in processed food salt and/or household salt.

Improvement of the dietary iodine-source partitioning approach should initially focus on reducing the individual variation of spot UIC and UNaC data. As demonstrated for UNaC data from NHANES [36] and INTERSALT studies [54] of US adults and also shown for UIC data from survey of school-age children [35], repeat urine collections from the same individuals in a subset of survey participants can be used to adjust the UIC and UNaC variables to more closely resemble their habitual intake distributions. The likely effect from adjusted UNaC data would be a less biased regression coefficient and, thus, more accurate estimates for native and food salt-source iodine intakes. Adjusted UIC data would reduce the uncertainty of all iodine intake point estimates. An additional improvement of technique could be achievable by using a native-source UNaC value in estimating the iodine intake sources.

Encouraged by the World Health Organization’s global action plan for the prevention and control of non-communicable diseases, many Governments have adopted a 30% reduction of mean population salt intake as target for 2025. For example, a protocol for national salt intake reduction has been developed in India and a baseline population salt intake estimate of 11 g per day has been reported [55]. The present approach of apportioning dietary sources of iodine intake may be useful to assist in striking a balanced choice between the two tactics of population salt intake reduction, namely salt-rich processed food reformulation and public education on the need to moderate the use of iodized household salt.

## 5. Conclusions

The results of this study were instructive by illustrating that, without any iodization of food-grade salt, the common diet in each country would fall short of sufficient iodine to meet population iodine requirements. Our study findings indicate major progress in Kenya towards ensured optimal iodine nutrition by universal supplies of properly iodized salt to food processers as well as households. In India, the study findings indicate impressive progress due to a strong national emphasis on universal supplies in consumer markets of good quality iodized household salt, which resulted in ample dietary iodine intakes in part by a default use of the same type of salt in mostly local preparation of food and meals outside the home. The findings in Senegal, in contrast, indicate that USI progress is seriously falling short. Starting out at the lowest native dietary iodine intake, the study findings in Senegal suggest that the additional, salt-derived iodine intakes are barely sufficient to raise the population iodine nutrition level above the minimum cut-off for iodine deficiency, due to SI contents in the salt supplies far below the regulatory standard and low household coverage of adequately iodized salt, particularly in rural areas. The study findings confirmed the existence of strong associations between the iodine and the sodium concentrations in single spot urine collections and, together with co-variation of the UIC values with SI contents in household salt, lend credibility to the importance of ensuring combined supplies of adequately iodized salt, both for processing of foods and for household use for sustained, equitable and optimum iodine nutrition in populations.

## Figures and Tables

**Table 1:** Descriptive characteristics of WRA ^1^ by country and household location.

	UIC (µg/L)		UnaC (mmol/L)		SI (mg/kg)	
	Median	IQR	Mean	SD	Mean	SD
Kenya (*n* = 382)						
Urban	187	203	204	91	38.0	18.4
Rural	164	161	177	102	32.3	17.9
All WRA Kenya	172	199	185	97	34.4	18.3
Senegal (*n* = 1110)						
Urban	116	132	168	76	18.1	13.0
Rural, salt producing	106	118	172	72	8.8	16.3
Rural, non-salt producing	76	104	165	85	10.4	10.6
All WRA Senegal	101	124	167	80	14.9	13.0
India (*n* = 2373)						
Urban	168	187	138	67	27.7	17.1
Rural	149	183	143	70	23.3	15.1
All WRA India	159	183	140	69	25.7	16.9

^1^ WRA = Women of reproductive age; UIC = Urinary iodine concentration; UNaC = Urinary sodium concentration; SI = Salt iodine.

**Table 2:** Associations of UIC ^1^ in WRA with UNaC, SI and other explanatory variables by country.

	Estimate ^2^	95% CI	*t*-Statistic	*p* Value
Kenya				
Intercept	4.112	3.477, 4.747	26.4	0.000
UNaC	0.0042	0.0034, 0.0050	10.5	0.000
SI	0.0056	0.0002, 0.0109	2.03	0.044
Rural areas ^3^	0.0436	−0.0171, 0.2585	0.40	0.690
Senegal				
Intercept	4.026	3.786, 4.267	33.2	0.000
UNaC	0.0021	0.0012, 0.0030	4.6	0.000
SI	0.0112	0.0053, 0.0170	3.8	0.000
Rural ^3^—salt processing	0.0655	−0.1395, 0.2705	0.63	0.528
Rural ^3^—non salt processing	−0.0736	−0.3009, 0.1537	−0.64	0.522
MPI—Education	0.0060	−0.2110, 0.2227	0.05	0.956
MPI—Health	−0.2699	−0.6605, 0.1207	−1.37	0.174
MPI—Living standards	−0.2018	−0.6345, 0.2328	−0.92	0.359
Bouillon consumption	0.0092	−0.0082, 0.0140	0.52	0.602
India				
Intercept	4.432	4.276, 4.588	55.8	0.000
UNaC	0.0024	0.0018, 0.0030	7.6	0.000
SI	0.0073	0.0032, 0.0114	3.5	0.001
Rural areas ^3^	−0.0532	−0.1531, 0.0467	−1.05	0.296
MPI—Education	0.0055	−0.1617, 0.1726	0.06	0.949
MPI—Health	0.0544	−0.1430, 0.2518	0.54	0.588
MPI—Living standards	−0.5141	−0.7477, −0.2806	−4.3	0.000

^1^ UIC = Urinary iodine concentration; UNaC = Urinary sodium concentration, SI = Salt Iodine, WRA = Women of reproductive age, MPI = Multi-dimensional Poverty Index score; ^2^ Weighted slope estimates are *β* coefficients from generalized linear regression with natural log-transformed UIC as dependent variable; ^3^ Compared to urban.

**Table 3:** UIC ^1^ portion estimates (µg/L; geometric mean, 95% CI) in WRA by country.

	Kenya (*n* = 382)		Senegal (*n* = 1110)		India (*n* = 2373)	
	Mean	95% CI	Mean	95% CI	Mean	95% CI
Native iodine	65	49, 82	56	46, 66	82	70, 94
Food salt	84	63, 104	24	15, 33	33	25, 40
Household salt	30	4, 56	12	6, 17	23	11, 35
Total UIC	179	160, 197	91	85, 98	138	131, 144

^1^ UIC = Urinary iodine concentration; WRA = Women of reproductive age.
